# Effectiveness of non-pharmaceutical measures in preventing pediatric influenza: a case–control study

**DOI:** 10.1186/s12889-015-1890-3

**Published:** 2015-06-09

**Authors:** Núria Torner, Núria Soldevila, Juan Jose Garcia, Cristian Launes, Pere Godoy, Jesús Castilla, Angela Domínguez, the CIBERESP Cases and Controls in Pandemic Influenza Working Group, Spain

**Affiliations:** Public Health Agency of Catalonia, Cr Roc Boronat 81-95, 08005 Barcelona, Spain; CIBER Epidemiología y Salud Pública, (CIBERESP), Cr de Casanova, 143, 08036 Barcelona, Spain; Public Health Department, Faculty of Pharmacy, University of Barcelona, Cr Roc Boronat 81-95, 08005 Barcelona, Spain; Pediatric Unit, Hospital Sant Joan de Déu, Cr Sant Joan de Déu, 2, 08950 Esplugues de Llobregat, Spain; Instituto de Salud Pública de Navarra, C/Leyre,15, 31002 Pamplona, Spain

**Keywords:** Influenza, Child, Non-pharmaceutical measures, Hand hygiene, Community setting, Prevention, Case control study

## Abstract

**Background:**

Hygiene behavior plays a relevant role in infectious disease transmission. The aim of this study was to evaluate non-pharmaceutical interventions (NPI) in preventing pediatric influenza infections.

**Methods:**

Laboratory confirmed influenza cases occurred during 2009–10 and 2010–11 seasons matched by age and date of consultation. NPI (frequency of hand washing, alcohol-based hand sanitizer use and hand washing after touching contaminated surfaces) during seven days prior to onset of symptoms were obtained from parents of cases and controls.

**Results:**

Cases presented higher prevalence of underlying conditions such as pneumonia [OR = 3.23; 95 % CI: 1.38 – 7.58 p = 0.007], asthma [OR = 2.45; 95 % CI: 1.17 – 5.14 p = 0.02] and having more than 1 risk factor [OR = 1.67; 95 % CI: 0.99 – 2.82 p = 0.05]. Hand washing more than 5 times per day [aOR = 0.62; 95 % CI: 0.39 – 0.99 p = 0.04] was the only statistically significant protective factor. When considering two age groups (pre-school age 0–4 yrs and school age 5–17) yrs , only the school age group showed a negative association for influenza infection for both washing more than 5 times per day [aOR = 0.47; 95 % CI: 0.22 – 0.99 p = 0.04] and hand washing after touching contaminated surfaces [aOR = 0.19; 95 % CI: 0.04 – 0.86 p = 0.03].

**Conclusion:**

Frequent hand washing should be recommended to prevent influenza infection in the community setting and in special in the school age group.

## Background

The community setting and hygiene behavior play an important role in controlling influenza, whether seasonal or pandemic, and emerging infectious diseases provide a good chance for promoting universal preventive measures such as hand hygiene [1]. The World Health Organization (WHO) and other health organizations have stressed the need to highlight the importance of non-pharmaceutical interventions (NPI) such as hand hygiene, social distancing and face mask as options to prevent influenza transmission in the community [2, 3]. Nevertheless, because some NPI measures are readily available and applicable for the general population they can be routinely instituted during influenza season regardless of pandemic threat evidence [4–7]. Household transmission of influenza can be reduced by the use of NPI, but low acceptability of some NPI such as facemasks make it more difficult to comply with some of these recommendations.

Hand hygiene is a key intervention for reducing transmission of acute respiratory infections (ARI) and other infections such as diarrhea in community settings. Hand hygiene has been specifically recommended for prevention of diseases with pandemic potential, such as severe acute respiratory syndrome and for pandemic influenza AH1N1pdm09. According to Aiello et al. improvements in hand hygiene resulted in reductions in respiratory illness by 21 %, hand-hygiene education with use of non-antibacterial soap, being the most beneficial intervention [7].

Yet studies examining hygiene practices during respiratory illness and interventions targeting aerosol transmission are needed [8]. It has been postulated that the effect of interventions may be higher in the pandemic scenario because of public anxiety giving way to higher rates of adherence, similar to what was observed during the SARS epidemic [9].

During 2009 influenza pandemic there was some uncertainty as to which NPI to recommend, especially whether facemasks should be endorsed [10].

The aim of the study was to investigate the effectiveness of non-pharmaceutical interventions in preventing cases of influenza in children in the community setting in 2009 pandemic and 2010–2011 post-pandemic/seasonal epidemic.

## Methods

A multicenter matched case–control study was performed in 7 regions of Spain from July 2009 to May 2011, with controls matched for age, hospital and date of hospitalization. For each confirmed hospitalized case of pandemic influenza, 1 confirmed outpatient case and 3 controls (2 hospitalized and 1 outpatient) were selected. Demographic variables, underlying medical conditions, use of antiviral agents, vaccines received and hygiene habits were collected for all cases and controls. The confirmed outpatient cases were obtained from the sentinel primary care Influenza surveillance network; of these only confirmed pediatric cases and controls which had been previously selected to match hospitalized influenza confirmed cases recruited for the multicenter case control study were used in this study [11]. All participating centers were granted approval by their ethics committees to carry out the study, namely the following: Comité Etico de Investigación Clínica de Euskadi; Comité Etico de Investigación Clínica Hospital Universitario Dr. Peset (Valencia); Comité Etico de Investigación Clínica Hospital Costa del Sol_Marbella (Màlaga); Comité Etico de Investigación Clínica Hospital Universitari Sant Joan de Déu de Esplugues (Barcelona); Comité Etico de Investigación Clínica Instituto Municipal de Asistencia Sanitaria_IMAS (Barcelona); Comité Etico de Investigación Clínica Dirección General de Salud Pública y Centro Superior de Investigación en Salud Pública (Madrid); Comité Etico de Investigación Clínica Comité Autonómico de ensayos clínicos de Andalucia and Comité Etico de Investigación Clínica Clínica de León and Corporación Sanitaria Parc Taulí de Sabadell.

### Settings

For each of the 7 regions where this study was performed the setting of recruited cases was the primary care sentinel pediatricians from the influenza surveillance network [12].

### Selection of cases and controls

Only Influenza AH1N1pmd09 virus was isolated in both influenza seasons studied. Pandemic season was considered from July 2009 to August 2010 when WHO declared the end of pandemic period] and post-pandemic or seasonal from October 2010 to May 2011. Outpatient children between 6-months and 17 years old with laboratory confirmed influenza and matched controls were recruited [12].

Size of the sample was calculated for the main study and the number of pediatric outpatient cases selected as outpatient controls for a hospitalized case made up the subset sample for this study on community based pediatric influenza cases and controls. Patients with influenza-like-illness (ILI) in whom influenza A H1N1pdm09 virus infection was confirmed by real time–polymerase chain reaction (RT-PCR) were selected from the outpatient Primary Care Influenza Surveillance Sentinel Network as cases. An outpatient control was selected from patients attending those same primary healthcare centers (PHC) consulting for other illness than ILI or other acute respiratory tract infection (ARI) Considering ILI symptoms as sudden onset of symptoms, fever and at least one of the following: malaise, headache, muscle pain; and at least one of the following: cough, sore throat, shortness of breath. Outpatient cases and controls were matched according to age (±3 years), geographical area and consultation date allowing for a range of ±10 days.

### Sociodemographic and clinical data

The following demographic variables and pre-existing medical conditions were collected for all study participants: age, sex, ethnicity, educational level and smoking habits of parents risk factors for acquiring influenza [history of pneumonia in the last two years, asthma, renal failure, diabetes, disabling neurological disease, neoplasia, transplantation, morbid obesity (BMI according to age) [13], treatment with systemic or inhaled corticosteroids, and antibiotic treatment within 90 days prior to onset of symptoms of the case and influenza (pandemic and seasonal)], [13]. Children were considered vaccinated with the seasonal influenza vaccine if they had received a dose of the vaccine at least 14 days before the onset of symptoms (cases] or the date of consultation for controls. For pandemic influenza vaccine children were considered vaccinated if they had received the pandemic influenza vaccine at least 7 days before the onset of symptoms (cases) or the date of consultation for controls. For the pneumococcal conjugate vaccines, a child was considered vaccinated if he had received the recommended doses according to age. Cases were considered vaccinated if they had received the last dose (or only dose if this was the schedule corresponding to their age) ≥14 days before symptom onset. Controls were considered vaccinated if they had received the last dose (or only dose if this was the schedule corresponding to their age) ≥14 days the date of consultation of the matched case. Information on the vaccination status was obtained from primary healthcare centre registers or vaccination cards.

### Information on and use of non-pharmaceutical interventions

A structured interview was used to determine whether study participants/parents had received information on preventing the transmission of pandemic influenza. Participants (or their parents if they were under 12 years) were asked about the use of non-pharmaceutical interventions as yes/no variables (frequent hand washing: 1–4 times/day >5 times/day, use of alcohol-based hand sanitizers, hand washing at home after touching potentially contaminated surfaces on public transport or in shops) and , frequency of public transportation use, and the use of face masks) seven days before the onset of symptoms in cases and controls. Frequency was asked for each variable and then broken down into two groups.

There were no refusals to participate in the study.

### Statistical analysis

A bivariate comparison was made between cases and controls for demographic variables and pre-existing medical conditions using Pearson’s Chi-square test for categorical variables and the Student *t* test for continuous variables. The crude Odds Ratio (OR) were estimated using the McNemar test.

A multivariate analysis was performed using conditional logistic regression to estimate adjusted OR (aOR), including age group (0–4 years and 5–17 years) and non-pharmaceutical interventions. Both seasons with circulating A H1N1pmd09 virus were also analyzed separately for the same variables. The analysis was performed using SPSS version 18.

### Data confidentiality and ethical aspects

All information collected was treated as confidential in strict observance of legislation on observational studies. The study was approved by the Ethics Committees of the participating hospitals. Written informed consent was obtained from patients included before interviews were carried out.

## Results

A total of 239 cases with confirmed influenza AH1N1pmd09 virus infection and 239 matched controls were included. Cases had a mean age of 5.4 years (SD = ±4.5), 42.3 % were females, 88.9 % were Caucasian, 74.3 % had secondary or higher education, 36.6 % had parents smokers and only 0.9 % had received any influenza vaccine while 35.9 % had received at least one dose of pneumococcal vaccine and 32.7 % were fully vaccinated according to their age. Controls had similar characteristics with no significant differences (Table 1).

**Table 1:** Distribution of sociodemographic variables and underlying medical conditions for confirmed outpatient cases and controls

	Cases	Controls	P value		Cases	Controls	Crude OR	P value
	(n = 239)	(n = 239)			(n = 239)	(n = 239)	(95 % CI)	
**Age. Mean (SD)**	5.4 ± 4.5	5.3 ± 4.6	0.48	**Pneumonia in previous 2 years**	23 (9.6 %)	8 (3.3 %)	3.23 (1.38 – 7.58)	0.007
**Age group**				**Asthma**	27 (11.3 %)	13 (5.4 %)	2.45 (1.17 – 5.14)	0.02
0-4	134 (56.1 %)	135 (56.5 %)	0.29	**Renal failure or nephritic syndrome**	4 (1.7 %)	2 (0.8 %)	2.00 (0.37 – 10.92)	0.42
5-17	205 (44.0 %)	104 (43.5 %)		**Diabetes**	1 (0.4 %)	2 (0.8 %)	0.50 (0.04 – 5.51)	0.57
**Female**	101 (42.3 %)	108 (45.2 %)	0.49	**Disabling neurological disease**	8 (3.3 %)	3 (1.3 %)	2.67 (0.71 – 10.05)	0.15
**Ethnicity**				**Transplantation**	4 (1.7 %)	1 (0.4 %)	4.00 (0.45 – 35.79)	0.21
White	209 (88.9 %)	218 (92.8 %)	0.56	**Obesity**	2 (1.1 %)	3 (1.6 %)	0.01 (0 – 1239.59)	0.44
Romany	9 (3.8 %)	5 (2.1 %)		**Previous antibiotics**	31 (13.0 %)	41 (17.2 %)	0.67 (0.38 – 1.18)	0.16
Amerindian	7 (3.0 %)	6 (2.6 %)		**Systemic corticosteroids**	10 (4.2 %)	4 (1.7 %)	3.20 (0.86 – 11.91)	0.08
Arab or North African	6 (2.6 %)	3 (1.3 %)		**Inhaled corticosteroids**	21 (8.8 %)	13 (5.4 %)	1.73 (0.82 – 3.63)	0.15
Other	4 (1.7 %)	3 (1.3 %)		**≥1 risk factors**	47 (19.7 %)	32 (13.4 %)	1.67 (0.99 – 2.82)	0.05
**Education level parents**				**≥2 risk factors**	7 (2.9 %)	3 (1.3 %)	2.52 (0.65 – 9.81)	0.18
Secondary or higher	168 (74.3 %)	160 (70.5 %)	0.35					
**Smoker parents**	87 (36.6 %)	98 (41.4 %)	0.36					

Cases presented a higher prevalence of underlying conditions such as pneumonia in the previous two years [OR = 3.23; 95 % CI: 1.38 – 7.58 p = 0.007], asthma [OR = 2.45; 95 % CI: 1.17 – 5.14 p = 0.02] and presenting more than 1 co morbidity [OR = 1.67; 95 % CI: 0.99 – 2.82 p = 0.05] (Table 1). The frequency of hand washing > 5 times [aOR = 0.62; 95 % CI: 0.39 – 0.99 p = 0.04] was the only statistically significant protective factor (Table 2).

**Table 2:** Distribution of health information and non-pharmaceutical measures for confirmed outpatient cases and negative controls

	Cases	Controls	Crude OR	P value	Adjusted OR^a^	P value
	(n = 239)	(n = 239)	(95 % CI)		(95 % CI)	
**Received information on influenza prevention**						
No	56 (23.6 %)	51 (22.0 %)	1		1	
Yes	181 (76.4 %)	181 (78.0 %)	0.83 (0.48 – 1.44)	0.51	0.72 (0.40 – 1.31)	0.29
**Frequency of daily hand washing**						
1-4 times	123 (52.3 %)	107 (45.7 %)	1		1	
>5 times	112 (47.7 %)	127 (54.3 %)	0.69 (0.45 – 1.04)	0.07	0.62 (0.39 – 0.99)	0.04
**Use of alcohol-based hand sanitizers**						
Never	158 (67.2 %)	170 (72.3 %)	1		1	
Sometimes	77 (32.8 %)	65 (27.7 %)	1.36 (0.85 – 2.17)	0.21	1.54 (0.8 – 2.66)	0.13
**Hand washing after touching contaminated surfaces**						
Never	50 (21.4 %)	43 (18.3 %)	1		1	
Sometimes	184 (78.6 %)	192 (81.7 %)	0.73 (0.41 – 1.32)	0.30	0.62 (0.29 – 1.31)	0.21
**Influenza vaccine**	2 (0.9 %)	5 (2.2 %)	0.44 (0.08 – 2.26)	0.32	0.27 (0.02 – 1.28)	0.09
**Pneumococcal vaccine**	79 (35.9 %)	69 (31.7 %)	1.52 (0.85 – 2.70)	0.15	1.47 (0.79 – 2.75)	0.22

^a^Adjusted for pneumonia in previous 2 years, asthma and disabling neurological disease

Statifying the sample into two age groups (0–4 years and 5 to 17 years) the only statistically significant risk factor was having had pneumonia in the previous two years [OR = 6.00; 95 % CI: 1.34 – 26.81 p = 0.02], was (Table 3).

**Table 3:** Distribution of medical conditions for confirmed outpatient cases and negative controls according to age group

	0-4 years				5-17 years			
	Cases	Controls	Crude OR	P value	Cases	Controls	Crude OR	P value
	(n = 122)	(n = 122)	(95 % CI)		(n = 93)	(n = 93)	(95 % CI)	
**Pneumonia in previous 2 years**	12 (9.8 %)	2 (1.6 %)	6.00 (1.34 – 26.81)	0.02	8 (8.6 %)	6 (6.5 %)	1.48 (0.47 – 4.70)	0.50
**Asthma**	10 (8.2 %)	4 (3.3 %)	3.00 (0.81 – 11.08)	0.10	16 (17.2 %)	9 (9.7 %)	2.07 (0.83 – 5.15)	0.12
**Renal failure or nephritic syndrome**	1 (0.8 %)	0 (0.0 %)	-	-	3 (3.2 %)	2 (2.2 %)	1.50 (0.25 – 8.98)	0.66
**Diabetes**	0 (0.0 %)	0 (0.0 %)	-	-	1 (1.1 %)	2 (2.2 %)	0.50 (0.04 – 5.51)	0.57
**Disabling neurological disease**	1 (0.8 %)	3 (2.4 %)	0.33 (0.03 – 3.20)	0.34	6 (6.5 %)	0 (0.0 %)	65.29(0.09– 46313)	0.21
**Transplantation**	0 (0.0 %)	0 (0.0 %)	-	-	4 (4.3 %)	0 (0.0 %)	65.29 (0.02 – 202501.6)	0.31
**Obesity**	0 (0.0 %)	1 (1.1 %)	-	-	2 (2.6 %)	2 (2.6 %)	0.01 (0 – 500061.3)	0.58
**Previous antibiotics**	18 (14.8 %)	27 (22.0 %)	0.50 (0.22 – 1.11)	0.09	9 (9.7 %)	11 (11.8 %)	0.83 (0.33 – 2.11)	0.69
**Systemic corticosteroids**	4 (3.3 %)	1 (0.8 %)	4.00 (0.45 – 35.79)	0.21	5 (5.4 %)	2 (2.2 %)	4.70 (0.52 – 42.78)	0.17
**Inhaled corticosteroids**	13 (10.7 %)	7 (5.7 %)	2.20 (0.76 – 6.33)	0.14	7 (7.5 %)	5 (5.4 %)	1.40 (0.44 – 4.41)	0.57
**≥1 risk factors**	16 (13.1 %)	12 (9.8 %)	1.50 (0.61 – 3.67)	0.37	29 (31.2 %)	18 (19.4 %)	1.97 (0.97 – 3.96)	0.06
**≥2 risk factors**	1 (0.8 %)	2 (1.6 %)	0.50 (0.04 – 5.51)	0.57	6 (6.5 %)	1 (1.1 %)	6.77 (0.81 – 56.84)	0.08

The school age group (5–17 years) showed a negative association for influenza infection for both washing more than 5 times per day [aOR = 0.47; 95 % CI: 0.22 – 0.99 p = 0.04] where in the 0–4 years group there was no significant association observed [aOR = 0.91; 95 % CI: 0.46 – 1.78 p = 0.77] and hand washing after touching contaminated surfaces [aOR = 0.19; 95 % CI: 0.04 – 0.86 p = 0.03] versus [aOR = 1.06; 95 % CI: 0.44 – 2.56 p = 0.77] in the 0–4 years group (Table 4)].

**Table 4:** Distribution of health information and non-pharmaceutical measures for confirmed outpatient cases and negative controls according to age group

	0-4 years					5-17 years				
	Cases	Controls	Crude OR	Adjusted OR^a^	P value	Cases	Controls	Crude OR	Adjusted OR^b^	P value
	(n = 122)	(n = 122)	(95 % CI)	(95 % CI)		(n = 93)	(n = 93)	(95 % CI)	(95 % CI)	
**Received information on influenza prevention**										
No	37 (30.6 %)	32 (26.0 %)	1	1		15 (16.3 %)	14 (15.9 %)	1	1	
Yes	84 (69.4 %)	91 (74.0 %)	0.71 (0.34-1.48)	0.74 (0.33 1.63)	0.45	77 (83.7 %)	74 (84.1 %)	0.83 (0.33 – 2.11)	0.72 (0.21 –2.50)	0.61
**Frequency of daily hand washing**										
1-4 times	57 (48.3 %)	54 (45.0 %)	1	1		58 (62.4 %)	42 (46.2 %)	1	1	
>5 times	61 (51.7 %)	66 (55.0 %)	0.80 (0.44- 1.47)	0.91 (0.46-1.78)	0.77	35 (37.6 %)	49 (53.8 %)	0.45 (0.23 – 0.87)	0.47 (0.22 – 0.99)	0.04
**Use of alcohol-based hand sanitizers**										
Never	84 (71.2 %)	96 (79.3 %)	1	1		62 (66.7 %)	62 (68.1 %)	1	1	
Sometimes	34 (28.8 %)	25 (20.7 %)	1.59 (0.85 -2.98)	1.45 (0.72 2.91)	0.29	31 (33.3 %)	29 (31.9 %)	1.09 (0.48 – 2.47)	1.29 (0.49 – 3.37)	0.60
**Hand washing after touching contaminated surfaces**										
Never	22 (18.8 %)	26 (21.5 %)	1	1		26 (28.0 %)	16 (17.6 %)	1	1	
Sometimes	95 (81.2 %)	95 (78.5 %)	1.36 (0.63-2.97)	1.06 (0.44-2.56)	0.90	67 (72.0 %)	75 (82.4 %)	0.27 (0.09 – 0.83)	0.19 (0.04 – 0.86)	0.03

^a^ Adjusted for: pneumonia in previous 2 years and previous antibiotics. ^b^ Adjusted for: frequency of daily hand washing and hand washing after touching contaminated surface

Stratifying the 0–4 age group into 0–2 years and 3–4 years, no significant differences were observed in cases and controls with respect to the questions referring to having received information regarding influenza prevention, frequency of daily hand washing or use of alcohol-based hand sanitizers or hand washing after touching potentially contaminated surfaces.

## Discussion

In the light of the possibility of other future influenza pandemics, examining public response to the A H1N1pmd09 influenza pandemic can provide information in terms of willingness to adopt preventive behaviors. In a polling study conducted by Steel Fisher et al. in five countries [14], in all countries the most commonly adopted personal protective behavior was to wash hands or use hand sanitizer more often.

This study highlights the effectiveness of hand washing in preventing infection due to influenza A H1N1pmd09 in the pediatric community setting. It has been proven by several authors that children at school age are a key factor in the spread of influenza epidemics [15–17]. This fact is underscored by several studies which have proven the effects of school closures on influenza outbreaks suggesting that school closure can reduce transmission of pandemic and seasonal influenza among schoolchildren, although the optimal school closure strategy is unclear [18].

Yet, although a large proportion of students may have adequate knowledge on influenza, only some of them practice preventive measures [19]. In our study we found a negative association for influenza infection for both washing more than 5 times per day and hand washing after touching contaminated surfaces in the school age group [5–17 years]. This fact stresses the importance of health education in which parents and teachers play an important role. No protective effect was observed in the multivariate analysis for the use of alcohol-based hand sanitizers. The use of masks could not be assessed due to the low number of responders to this question in the pediatric outpatient setting. Although there is limited data to support the use of face masks and/or respirators in healthcare and community settings, evidence suggests that given the potential loss of effectiveness with incorrect usage, general advice should be to only use masks/ respirators under very particular, specified circumstances, and in combination with other personal protective practices [20]. Other studies in our setting have found an adherence rate of < 50 %, and suggested that facemasks are not useful in reducing seasonal influenza infections in the community due to their low acceptance, although they could be effective in those who wore them [11].

Yet other authors find there is evidence to support the use of masks and/or respirators in healthcare or community settings [21]. Mask use is best undertaken as part of a package of personal protection, especially including hand hygiene in both home and healthcare settings. However, examination of the literature has shown that most of the studies examined were too small to reliably detect what would be anticipated to be moderate effects [22].

Frequency of public transportation use was included despite no recommendation was made by the Spanish health authorities to avoid using public transportation during the pandemic, because it can be considered a mass gathering site and a source of infection transmission.

In our study only 0.9 % of the children with underlying chronic medical conditions, who were eligible for vaccination, had received any influenza vaccine, a fact that was also observed by Altmann et al. They showed that 93 % of these children, had not been vaccinated [23]. Low vaccination coverage among children aged <5 years who are at greatest risk for influenza associated complications, and the low coverage among older children and adolescents suggest that continued implementation of existing strategies and the development of new strategies to improve seasonal vaccination coverage are needed [24]. The significant burden of influenza, on children should encourage upgrading vaccination coverage in these age groups and especially for those included in risk groups for whom yearly vaccination is recommended [25, 26].

As other authors have underscored, our data also showed that clinical and epidemiological characteristics of children infected with pandemic H1N1 2009 influenza A versus those infected with post-pandemic/seasonal influenza AH1N1pmd09 [27, 28] bear no significant differences. For both seasons, significant differences were shown for cases having presented with pneumonia in the previous two years, asthma, inhaled corticosteroid treatment and presenting with more than 1 co-morbidity.

There are several limitations to the study which have to be considered, especially because of possible recall bias with respect to report of hand-washing behavior among cases and controls, although the questionnaires were completed within few days from consultation. There can also be a social desirability bias because of the fact that people might say they do wash their hands or use a hand sanitizer because that is what they are suppose to do. This may carry a certain misclassification of respondents which can not be accurately assessed.

Basic infection prevention interventions, including hand hygiene [using soap and water or alcohol-based hand sanitizers), have been shown to reduce the spread of respiratory viruses, particularly among young children [29]. Stebbins et al. have shown that a respiratory hygiene intervention that included hand sanitizer use reduced school absences related to influenza A. Although no significant effect of the intervention on the primary study outcome of all laboratory-confirmed influenza cases was found, there were statistically significant differences in protocol-specified ancillary outcomes. Children in intervention schools had significantly fewer laboratory-confirmed influenza A infections than children in control schools [30].

Allison et al. concluded that hand gel use is a feasible strategy in settings were frequent hand washing might not be possible, such as in elementary schools, and even though acceptance of facemask use was low, most teachers stated they would use masks in their classroom in a pandemic [31]. Evidence from household, as well as institutions, demonstrate that personal hygiene continue to be a key prevention strategy against infection, thus it is necessary to stress the importance of keeping proper hygiene and cleaning practices to reduce the risk of spreading infection [32].

## Conclusion

The success of an NPI or a set of NPIs depends on both its efficacy and the feasibility of implementing it with relevant populations. If NPIs are to be used in community settings during a severe influenza season or pandemic, to increase acceptability on the part of the adults is of utmost importance. Without such acceptance, it is highly unlikely that children and their supervising adults will participate [33].

This study emphasizes the importance of simple infection prevention measures, such as hand hygiene which have been proven effective to reduce the transmission of infection in schools and the spread to household contacts by Papenburg J,et al. [34]. Frequent hand washing should be recommended to prevent influenza infection in the family and community setting and in special in the school age group.
